# A Human Ovarian Tumor & Liver Organ-on-Chip for Simultaneous and More Predictive Toxo-Efficacy Assays

**DOI:** 10.3390/bioengineering10020270

**Published:** 2023-02-18

**Authors:** Arianna Fedi, Chiara Vitale, Marco Fato, Silvia Scaglione

**Affiliations:** 1 Department of Computer Science, Bioengineering, Robotics and Systems Engineering (DIBRIS), University of Genoa, 16126 Genoa, Italy; 2 National Research Council of Italy, Institute of Electronic, Computer and Telecommunications (IEIIT), 16149 Genoa, Italy; 3 Department of Experimental Medicine (DIMES), University of Genoa, 16126 Genoa, Italy; 4 React4life S.p.A via Fiasella 1, 16121 Genova, Italy

**Keywords:** 3D in vitro models, multi-organ, drug efficacy, fluid-dynamics, organ-on-chip, ovarian cancer, drug diffusion

## Abstract

In oncology, the poor success rate of clinical trials is becoming increasingly evident due to the weak predictability of preclinical assays, which either do not recapitulate the complexity of human tissues (i.e., in vitro tests) or reveal species-specific outcomes (i.e., animal testing). Therefore, the development of novel approaches is fundamental for better evaluating novel anti-cancer treatments. Here, a multicompartmental organ-on-chip (OOC) platform was adopted to fluidically connect 3D ovarian cancer tissues to hepatic cellular models and resemble the systemic cisplatin administration for contemporarily investigating drug efficacy and hepatotoxic effects in a physiological context. Computational fluid dynamics was performed to impose capillary-like blood flows and predict cisplatin diffusion. After a cisplatin concentration screening using 2D/3D tissue models, cytotoxicity assays were conducted in the multicompartmental OOC and compared with static co-cultures and dynamic single-organ models. A linear decay of SKOV-3 ovarian cancer and HepG2 liver cell viability was observed with increasing cisplatin concentration. Furthermore, 3D ovarian cancer models showed higher drug resistance than the 2D model in static conditions. Most importantly, when compared to clinical therapy, the experimental approach combining 3D culture, fluid-dynamic conditions, and multi-organ connection displayed the most predictive toxicity and efficacy results, demonstrating that OOC-based approaches are reliable 3Rs alternatives in preclinic.

## 1. Introduction

In medical and pharmaceutical research, there is an urgent challenge related to the evident and increasingly low rate of successful research results being translated from bench to bedside [1,2,3]. In particular, there is a disproportion between the huge investments in new medicines and their impact on population health [4] since about 90% of early clinical trials fail [5,6,7]. This problem is particularly significant in oncology, where the statistics are more dramatic [7,8].

The main reason for this failure is related to the lack of clinical efficacy and unmanageable organ toxicity [9,10], and these poor predictive results are often attributed to the poor ability of preclinical models to generate results of human relevance [7,11,12,13,14,15]. Indeed, current 2D models are far from representative of human complexity, and while emerging 3D models more closely resemble chemical and biomechanical environmental in vivo-like cues [16], they are still insufficient in recapitulating human disease progression and tissue–drug dynamic interaction [17,18,19,20,21]. On the other hand, while animal tests are required by regulatory organizations, playing a pivotal role in evaluating the safety and efficacy of drugs [6,22], there is still a severe mismatch in the diversity of animal species that explain this poor animal-to-human predictability. As a result, less than 8% of animal trials are successfully translated to clinical cancer trials [1,11,23,24,25,26,27,28,29,30]. Indeed, animal studies have been demonstrated to overestimate the likelihood of a treatment being effective by about 30%, while toxic outcomes at sub-clinical and lower doses than those found to be safe in animals have been reported [31,32]. Hereafter, although in vivo models continue to provide useful information about drug safety and potency, it is crucial to keep in mind that their findings are rarely applicable to humans [1,6,33,34].

To circumvent these shortcomings, alternative human-relevant approaches have emerged, the most significant being organ-on-chip (OOC) systems [35]. These technologies are capable of accurately decoupling biological mechanisms behind the cytotoxic effects of testing molecules through a highly reproducible and less time- and cost-consuming in vitro approach. In particular, it is hoped that OOCs will advance biological contexts and thus potentially produce physiologically-relevant outcomes translatable to clinical scenarios [20,36,37,38]. OOC systems exploit the advances made in the last decade in microfabrication and biomimicry of materials, as well as novel methodologies for using primary cells or patient-derived biopsies in vitro [35,39,40,41]. These unprecedented multidisciplinary combinations allow the recapitulation of the functionality, architecture and dynamics at the organ level, setting the stage for more efficient preclinical predictions of human responses and thus potentially diminishing the high attrition rates of clinic trials [42]. However, many human diseases, such as cancer, involve multiple organs, and thus 2D static models used to manage spatiotemporally separated organs are too simplistic and fail to simulate the dynamic interrelated physiology of tissues and their interaction with the tested drugs [43,44]. Indeed, tissues and cells within the body are anatomically connected by a network of vessels, where fluids flow and communicate by the secretion of signaling factors (e.g., extracellular vesicles, soluble molecules) [40]. Hence, organ-organ interplays are crucial to our understanding of human disease and they should also be introduced to model and achieve appropriate responses to investigational drugs. Moreover, it is well known that drugs may cause multi-organ side effects and when they enter the human body, they are bio-transformed (e.g., ADME processes) [30,43]. Thus, to properly emulate the pharmaco-kinetics and pharmacodynamics of drugs, systemic approaches must be pursued. Specifically, multi-organ-on-chip (MOOC) systems have been recently developed to address this need, reproducing the multicellular nature of organs and their connections, which would not have been possible to implement in single OOCs [43,45]. Particularly, multi-organ platforms with medium recirculation emulate systemic and cross-organ communication and reciprocal influences, better reproducing the in vivo context [39,43]. For example, MOOCs incorporating a liver model may bring essential information about hepatic metabolism and the hepatotoxicity of the compounds tested, as has been reported for many conventional therapies [46,47]. Moreover, MOOCs can be valuable tools for developing metastasis models for the online monitoring of cancer cells extravasation from the primary mass and their circulation and intravasation into one or multiple specific metastatic sites [44,47].

However, although they are promising tools in cancer research, these approaches are currently based on miniaturized cell culture, allowing the adoption of a small number of cells, which may not accurately represent phenotypic or microenvironmental characteristics or tiny tumor samples, thus limiting the use of analytic techniques [17,20]. Moreover, fluid flow is mostly driven by gravity or capillary forces, preventing important fluidic cues, such as velocity and shear forces of the human bloodstream, from modulating through a pumping system [17,48]. Finally, both conventional and advanced microfluidic devices are made of polydimethylsiloxane (PDMS), which is known to be potentially harmful to cells by causing biocompatibility loss due to the gradual release of non-crosslinked oligomers and the adsorption of small hydrophobic molecules [20,48,49].

In this study, to overcome these limitations, we adopted a multicompartmental PDMS-free OOC technology (MIVO^®^) for testing the efficacy of on-target and simultaneously measuring off-target toxicity, determining the therapeutic index in a pathophysiological scenario. The system enables the combination of 3D clinically relevant-sized tumor tissue cultures with a fluidic circuit controlled by a peristaltic low flow rate pump that accurately mimics the capillary bloodstream, allowing systemic drug administration to be simulated. Specifically, a 3D hydrogel-based ovarian cancer model [17] was generated and fluidically connected to a hepatocellular culture; cisplatin was injected in circulation and the anti-cancer effects and hepato-toxicity were assessed after 48 h of treatment, respectively. Computational fluid-dynamic (CFD) simulations have been performed to (i) set capillary-like blood flow dynamics and (ii) investigate the mass transport profiles of cisplatin within the cell culture models. Finally, toxo-efficacy results derived from the dynamic multi-organ system were compared with (i) static single-organ models, (ii) dynamic single-organ models, and (iii) static multi-organ models.

## 2. Materials and Methods

### 2.1. Liver and Ovarian Cancer Cell Cultures

The human liver HepG2 cell line (ATCC No. HB8065) was expanded in Eagle’s Minimum Essential Medium (EMEM), supplemented with 10% heat-inactivated Fetal bovine serum (FBS), 2 mM L-glutamine (L-glu), and 1% penicillin/streptomycin (Pen/Strep), and plated at a density of 1 × 10^5^ cells/cm^2^. The human ovarian cancer SKOV-3 cell line (ATCC) was expanded Dulbecco’s modified Eagle’s medium (DMEM) high glucose supplemented with 10% FBS, 2 mM L-glu and 1% Pen/Strep and plated at a density of 1 × 10^5^ cells/cm^2^. The cells were incubated in a humified, 5% CO_2_ atmosphere at 37 °C. Media were changed twice a week. When culture dishes were nearly confluent, liver, and ovarian cancer cells were detached with 0.25% trypsin-EDTA solution, after two washes with Phosphate Buffered Saline (PBS) and replated until the next confluence. FBS, L-glu, Pen/Strep solution, DMEM, EMEM, trypsin-EDTA solution, trypsin solution, Live/Dead assay, Phalloidin Fluorescein Isothiocyanate Labeled, DAPI, and cisplatin were purchased from Sigma Aldrich.

A first set of cells culture in 2D was adopted to preliminary investigate the cytotoxic effect of cisplatin on HepG2 and SKOV-3 that were cultured as monolayers at a density of 1 × 10^5^ cells/mL in 96-well plates. Cells were left to adhere overnight in a humified, 5% CO_2_ atmosphere at 37 °C. The day after (T0), cisplatin was added to the culture medium at a final concentration of 10, 25, 50, and 100 μM.

### 2.2. Ovarian Cancer Model

A 3D hydrogel-based ovarian cancer model was developed as in our previous publication [17]. Briefly, Alginate powder (React4life) was dissolved in physiologic solution at 1% *w/v* and filtered under sterile conditions. SKOV-3 cells suspension was mixed with the alginate solution to obtain a final concentration of 0.5% *w/v*. The SKOV-3/alginate suspension was dripped into a sterile 0.5 M Calcium-based crosslinker bath to form hydrogel spheres with a final density of cells of 1 × 10^6^ cells/mL. After washing the spheres with DI water to remove the excess of calcium, the hydrogels were gently moved into the culture systems, placed in 96-well plates, and cultured with DMEM supplemented with 10% FBS, 1% Pen/Strep, and CaCl_2_ 5 mM. The day after (T0), cisplatin was added to the culture medium at a final concentration of 10, 25, 50, and 100 μM.

### 2.3. Static vs. Dynamic In Vitro Model

A co-culture model was implemented by cultivating SKOV-3 cells within 24-well Transwell inserts accommodated within the 24-well plates where HepG2 cells were plated on the bottom. Cells were left to adhere overnight in a humified, 5% CO_2_ atmosphere at 37 °C. The day after (T0), cisplatin was added to the culture medium at a final concentration of 10, 25, and 50 μM.

A compartmental fluidic device (MIVO^®^ by React4life) was adopted to simultaneously assess the on-target and off-target cytotoxic activity of cisplatin under dynamic conditions. Briefly, inserts containing either HepG2 cells or SKOV-3 cancer spheres were placed within the MIVO^®^ chamber, producing two fluidically independent compartments: (i) the tissue culture chamber, and (ii) the circulatory one.

The device was connected to a pumping system imposing a fluid flow predicted by CFD simulations through a closed-loop fluidic circuit that mimics the circulatory system disseminating the drug. Cisplatin was injected into the fluidic circuit, resembling systemic drug administration and extravasation.

For the dynamic multi-organ condition, two independent chambers hosting liver and ovarian cancer models were fluidically connected by the external fluidic circuit, reproducing the in vivo-like tissues arrangement and interconnections. Mono-culture or co-culture of HepG2 cells and SKOV-3 cells provided with the same amount of culture medium with or without cisplatin were used as static controls.

### 2.4. Computational Fluid-Dynamic Analyses

Fluid dynamics within the organ-on-chip was investigated to predict (i) the fluid velocity profiles within the device, and (ii) the transport kinetics of cisplatin, as the anticancer drug tested. First, the analysis was performed by using the Single-Phase Laminar Fluid Flow model of Comsol Multiphysics 5.6, assuming (i) a laminar flow regime and (ii) an incompressible Newtonian fluid. The equations to be solved include Navier–Stokes for the conservation of momentum (1a) and the continuity law for the conservation of mass (1b):

$$\left\{ \begin{array}{cc} \rho \left[\frac{\partial u_{f}}{\partial t}+u_{f}\cdot \left(\nabla u_{f}\right)\right]=-\nabla p+\mu \left(\nabla^{2}u_{f}\right) & (1a)\\ \left(\nabla \cdot u_{f}\right)=0 & (1b) \end{array}\right.$$

where ***u_f_*** is the fluid velocity, and p is the pressure across the circuit. The values of the density ρ (1000 kg/m^3^) and the viscosity *μ* (10^−3^ Pa·s) were selected as water at room temperature (37 °C). A flow rate of 2 mL/min was set as input according to the value imposed experimentally to generate the fluid motion, whereas as output the atmospheric pressure was set as null, avoiding a backflow. A no-slip boundary condition was set. Finally, an iterative geometric multigrid (GMRES) algorithm was used to solve the equations.

Subsequently, cisplatin mass transport analysis was carried out by using the Transport of the diluted species (TDS) module of Comsol Multiphysics. In addition to the diffusive mechanism, an additional two processes were considered: the convection transport, due to the presence of a velocity field (**u_f_**), and the metabolite and drug consumption due to cellular activity. The reaction term R for the drug was defined according to the Michaelis–Menten kinetics:

(2) $$R=\frac{V_{max}\;c}{K_{m}+c}\;\;\;\;$$

where *c* is the concentration of the component, *V_max_* represents the maximum consumption rate (equal to 1.66 × 10^−12^ mol/m^3^·s for cisplatin) and *K_m_* represents the component concentration when the rate is *V_max_*/2 (equal to 6.64 × 10^−3^ mol/m^3^ for cisplatin) [17,19]. Thus, the general form to describe mass transport can be written as:

(3) $$\frac{\delta c}{\delta t}+\nabla \cdot \;\left(J\right)+u_{f}\;\cdot \;\nabla c=R$$

where *c* is the component concentration and ***J*** is the mass diffusive flux vector, defined by Fick’s law:

(4) J=−D ∇c

where *D* is the diffusion coefficient of the molecule, previously reported in the literature [17,19].

This study was conducted by considering 2D and 3D alginate hydrogel-based culture scenarios to monitor the transport profiles of the compounds over time, both in static and dynamic conditions, with the same culture medium volume. In both cases, 0.1 mol/m^3^ was set as the initial culture medium concentration for cisplatin [19].

### 2.5. Cell Viability and Pharmacodynamic Evaluation

Cell viability and proliferation of both HepG2 and SKOV-3 were quantitatively assessed through Alamar Blue Assay. After 48 h of drug treatments, culture media were changed with fresh medium containing 10% *v/v* of Alamar Blue solution. Samples were incubated at 37 °C for 4 h in the dark, the supernatants were collected, and absorbance was measured spectrophotometrically (Infinite 200 Pro). Cell viability was calculated as the percentage of live cells normalized to the untreated controls. The proliferation rate was derived as the ratio between the time point of interest and the number of cells at T0.

A pharmacodynamic evaluation was performed by calculating the cisplatin half maximal effective concentration (EC50) for SKOV-3 and median lethal dose (LD50) for HepG2 cells under different culture conditions. These parameters were estimated as best-fit values of a non-linear fitting dose-response model by GraphPad Prism software (La Jolla, CA, USA).

To investigate possible SKOV-3 and HepG2 cells crosstalk, a conditioned media experiment was carried out. Briefly, a conditioned culture medium was produced by culturing HepG2 cells as monolayers and SKOV-3 cells as monolayers or embedded in a 3D alginate hydrogel with or without cisplatin (10, 25, 50 μM) for 48 h. After, this culture medium was collected and administered to the other cell type (i.e., culture medium collected from HepG2 cells was administered to SKOV-3 cells and vice versa) to evaluate the possible impact on the cellular growth rate. HepG2 and SKOV-3 cells cultured with fresh culture medium were used as the negative control.

### 2.6. Immunofluorescence

SKOV-3 viability, both cultured as monolayers and embedded within alginate, was qualitatively evaluated through live/dead assay. Briefly, after 48 h of treatment, samples were washed with PBS and incubated in 2 mM Calcein-AM and 4 mM EthD-1 in PBS for 15 min at 37 °C in a dark environment to detect live and dead cells, respectively. Then, samples were washed three times in PBS and observed by means of fluorescence microscopy (Nikon H550L).

HepG2 clusters disaggregation and morphological changes upon cisplatin treatments were evaluated by visualizing cytoskeleton alteration with Phalloidin Fluorescein Isothiocyanate Labeled. Briefly, cells were fixed with 4% paraformaldehyde in PBS (PFA; pH 7.4) for 15 min and permeabilized with 0.1% Triton X-100 for 5 min. Subsequently, cells were incubated with 1% bovine serum albumin (BSA). Then, cells were incubated with Phalloidin Fluorescein Isothiocyanate Labeled (1:5 in DI) for 1 h at RT. Nuclei were counter-stained with DAPI. Then, samples were washed three times in PBS and imaging was performed by using fluorescence microscopy (Nikon H550L).

All images obtained were analyzed through ImageJ software.

### 2.7. Statistical Analysis

Student’s paired t-test between each drug treatment condition and the respective control was performed at specific time points; statistical significance was set at *p* < 0.05 and *p* < 0.001.

## 3. Results

### 3.1. Static Cisplatin Toxo-Efficacy Evaluation

#### Mono-Culture vs. Co-Culture Conditions

**Figure 1:**
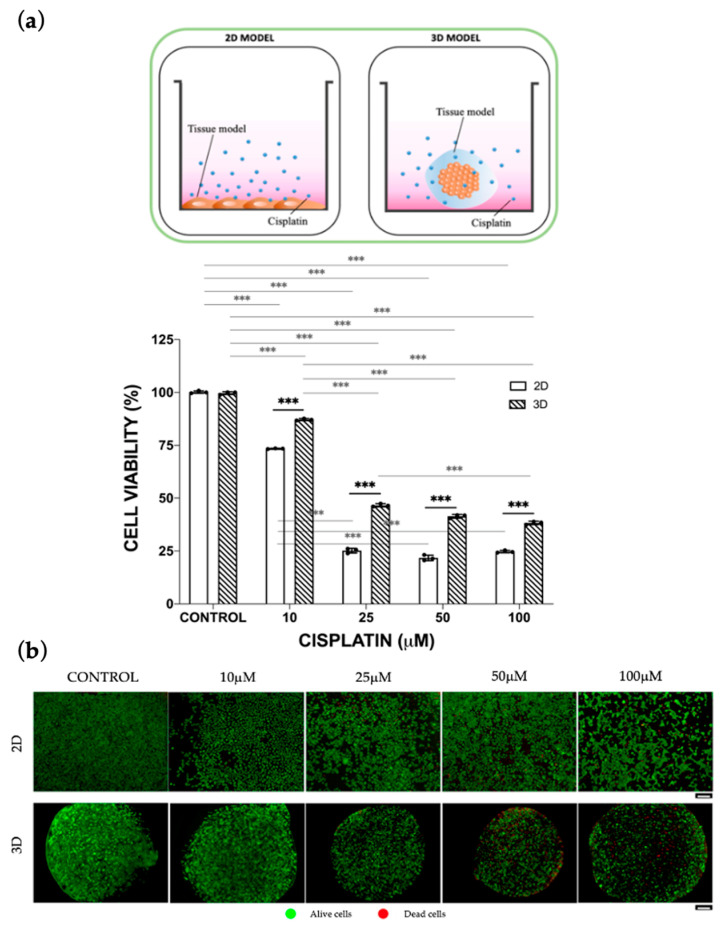
Cisplatin efficacy after 48 h of treatment. (**a**) SKOV-3 viability was assessed by Alamar Blue assay both in 2D and in 3D static mono-culture models treated with the 10, 25, 50, 100 μM cisplatin concentrations. Cell viability was derived as the percentage of alive cells normalized to the untreated controls. Values are reported as mean ± SD. Student’s paired t-test between each experimental condition was performed and statistical significance was set at *** *p* < 0.001 (N = 3 biological replicates; *n* = 3 technical replicates). (**b**) Cell viability upon cisplatin treatment represented by live/dead assay. Scale bar is 100 μm.

A screening of cisplatin concentrations was conducted to investigate both the efficacy and toxicity on ovarian cancer and liver cells, respectively. Regarding the cancer model, both the 2D and the 3D models were investigated, both in monoculture and co-culture. For mono-culture efficacy assays, Skov-3 cells were grown in 2D monolayers or embedded within 3D alginate-based spheres [17] and treated with cisplatin (10 μM, 25 μM, 50 μM and 100 μM) for 48 h. As expected, the percentage of alive cells decreased with increasing cisplatin concentrations, and it was significantly higher in 3D models than in 2D. Consequently, the derived EC50 parameter shifted from 15 μM (2D model) to 32.5 μM (3D model), showing a higher drug resistance of cells when they are surrounded with an extracellular matrix (ECM)-mimicking matrix (Figure 1).

**Figure 2:**
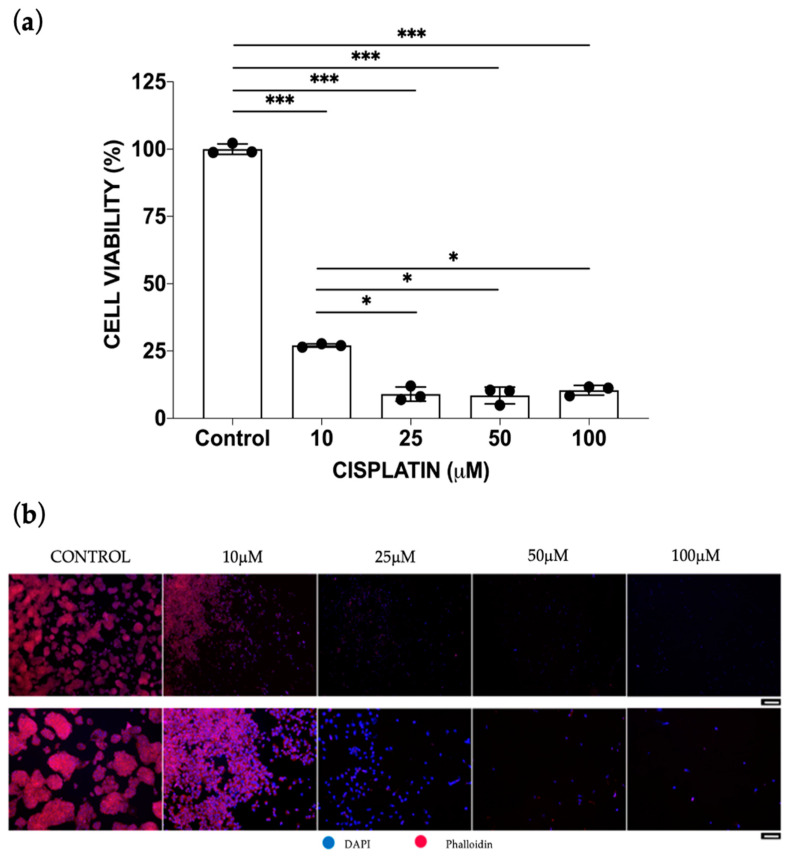
Cisplatin toxicity after 48 h of treatment. (**a**) HepG2 viability was assessed by Alamar Blue assay in 2D static mono-culture models treated with the 10, 25, 50, 100 μM cisplatin concentrations. Cell viability was derived as the percentage of alive cells normalized to the untreated controls. Values are reported as mean ± SD. Student’s paired t-test between each experimental condition was performed and statistical significance was set at * *p* < 0.05 and *** *p* < 0.001 (N = 3 biological replicates; *n* = 3 technical replicates). (**b**) Images of nuclei (DAPI) and cytoskeleton (Phalloidin) of HepG2 cells upon cisplatin treatment. Scale bar is 100 μm.

For the mono-culture toxicity assays, the same concentrations of cisplatin were administered to HepG2 cell monolayers. As can be seen from Figure 2a, cisplatin was revealed to be highly toxic for HepG2 cells, with a remarkable reduction in cell viability up to 25% that was detected at the lowest cisplatin concentration (10 μM). The LD50 parameter was 4 μM. Interestingly, the cisplatin cytotoxic activity against HepG2 cells was also shown through a DAPI-Phalloidin staining: an increasing disaggregation of typical HepG2 cell clusters was noted with the increase in drug doses, indicating that this anticancer drug leads also to a cytoskeletal destructive effect on actin filaments (Figure 2b).

**Figure 3:**
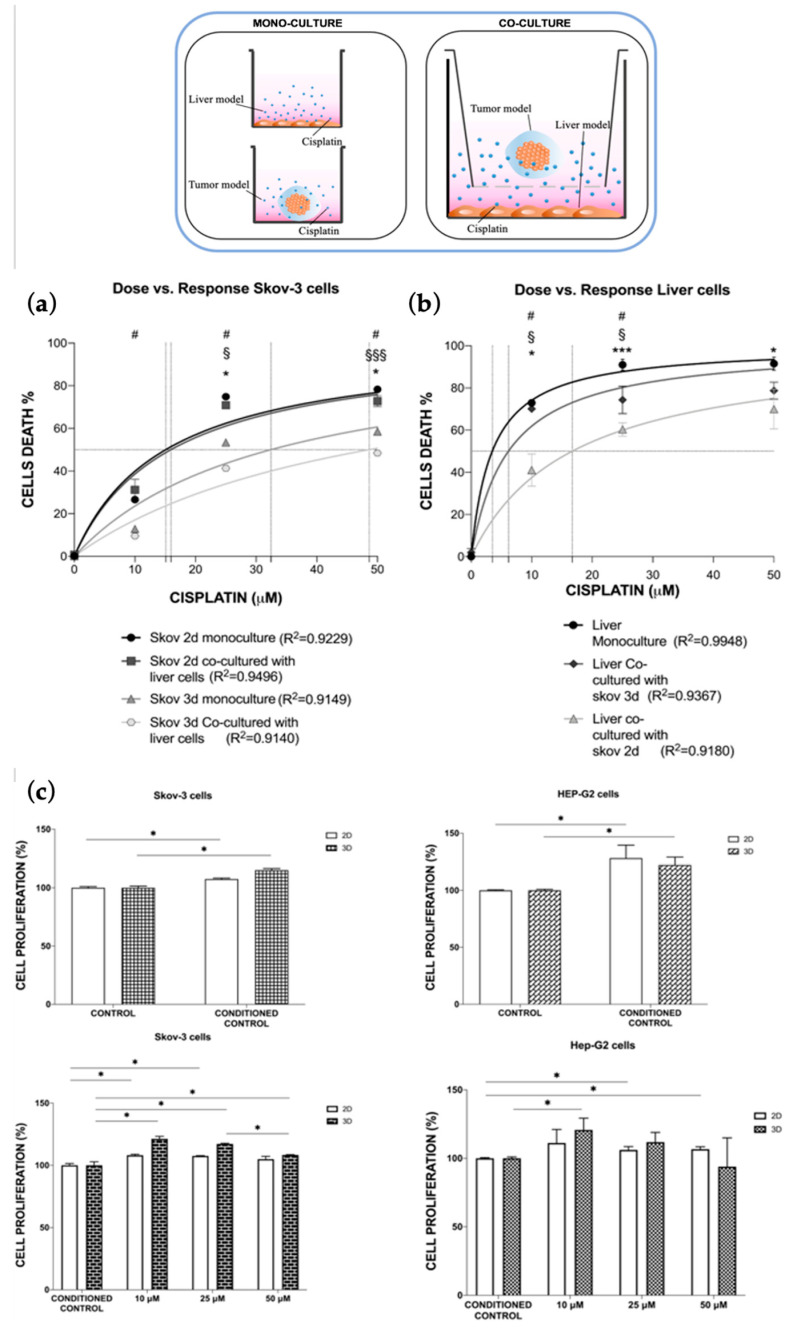
Cisplatin response in mono-culture vs. co-culture conditions and conditioned media effect. Cisplatin dose-response of SKOV-3 (**a**) and HepG2 (**b**) cells under different culture conditions. Values are reported as mean ± SD. For each condition, the EC50 (vertical dotted lines, left image) and LD50 parameters (vertical dotted lines, right image) were calculated as best-fit values of a non-linear fitting dose-response model (solid curves) by GraphPad Prism software. Statistical significance was set at *, §, # *p* < 0.05 and ***, §§§ *p* < 0.001 (N = 3 biological replicates; *n* = 3 technical replicates). (**c**) SKOV-3 (**left**) and HepG2 (**right**) cell proliferation were assessed after the conditioned media administration. Control is the negative control (i.e., cells that received fresh culture medium); conditioned control: cells with culture medium w/o cisplatin previously conditioned with the other cell type for 48 h; 10, 25, 50 μM are values of cells with conditioned culture medium with cisplatin. Statistical significance was set at * *p* < 0.05 and *** *p* < 0.001 (N = 3 biological replicates; *n* = 3 technical replicates).

A co-culture of HepG2 and SKOV-3 cells was then realized to evaluate whether the co-presence of multiple tissue models could affect a single cell-type response to cisplatin. SKOV-3 cells grown in 2D monolayers or embedded within 3D alginate-based spheres were cultured onto porous permeable inserts and placed above HepG2 cell monolayers (Figure 3). Cisplatin directly added to the culture medium generated a significant dose-dependent inhibition in cell viability for both cell lines as occurred for mono-cultures. Intriguingly, considering the 10 μM dose (Figure S1), which is in line with cisplatin plasma concentrations observed in clinics [50], the hepato-toxicity in the co-culture model was reduced in respect to the mono-culture condition. On the other hand, the ovarian cancer cells displayed significantly higher drug resistance when encapsulated in 3D alginate hydrogels, whereas their viability in 2D cultures was reduced when cancer cells were co-cultured compared to the mono-culture 2D, showing a more significant effect of the cell culture dimensionality (2D vs. 3D) than the presence of a concurrent cellular model (mono-culture vs. co-culture).

To further inspect cell interplays and tissue crosstalk, conditioned media experiments were carried out: a higher proliferation rate was observed for Skov-3 (107% and 115%) and HepG2 (128% and 122%) cells fed with a drug-free medium—previously used for culturing the other cell type—than those that received fresh culture medium (Figure 3c), pointing out the possible presence of soluble signaling molecules (e.g., cytokines) released by the cells during the first culture that stimulates the growth of the second ones, and thus the occurrence of cell interactions. Furthermore, this phenomenon was more noticeable when cisplatin was present in the conditioned medium in a dose-dependent manner; intriguingly, enhanced cell proliferation was identified with respect to the case of drug-free medium treatment, with a decreasing trend as the administered concentration increased. Particularly, both SKOV-3 and HepG2 cells in all culture configurations displayed a higher proliferation rate (108% and 121% for SKOV-3, 11% and 120% for HepG2) when cisplatin was administered at 10 μM as the initial concentration. This could suggest that further and/or diverse mechanisms of cell-cell communication and protection occur when cisplatin induces apoptosis.

### 3.2. Dynamic In Vitro Cisplatin Toxo-Efficacy Evaluation

#### 3.2.1. Fluid-Dynamic and Mass Transport Analysis

**Figure 4:**
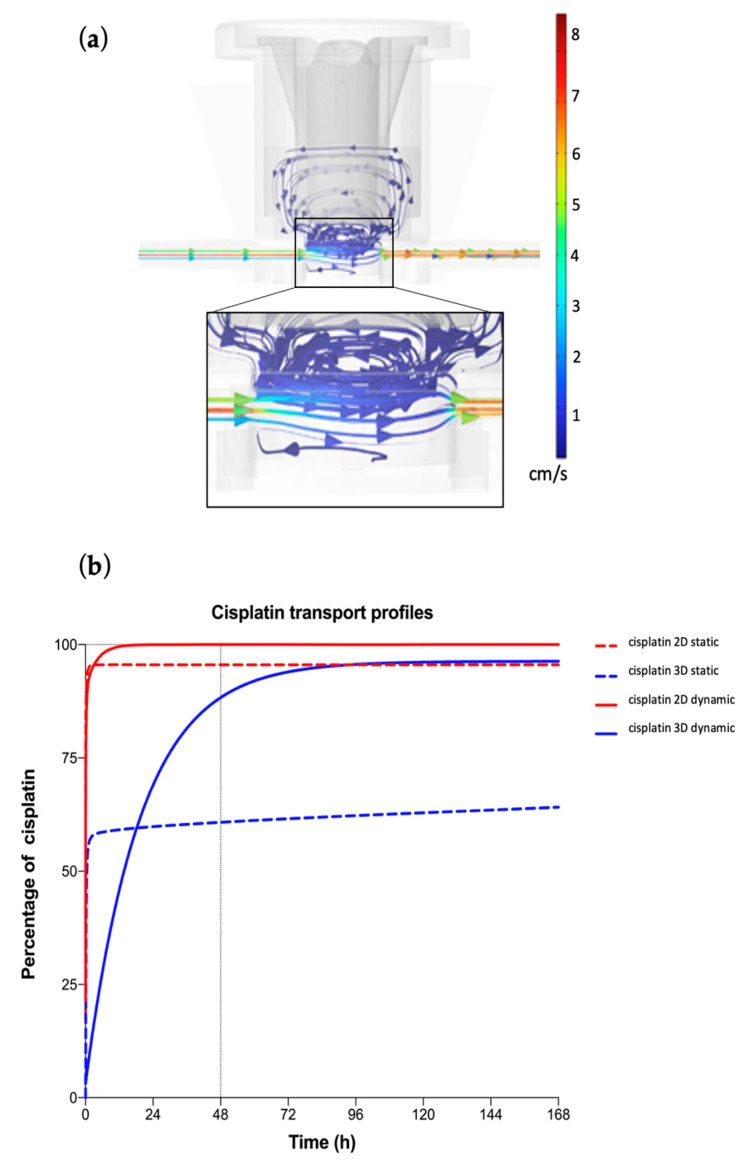
CFD simulations. (**a**) CFD streamlines illustrating the flow field of the circulating fluid within the MIVO^®^ chamber in terms of velocity profile when a flow rate of 2 mL/min is imposed. (**b**) Molecule mass transport was derived up 7 days by using the TDS module of Comsol Multiphysics. The percentage of the cisplatin molecule that is indicated on the ordinate axis was calculated as the ratio between the concentration measured at the center of the considered model (2D cell monolayer or 3D hydrogel sphere) and the initial concentration in the culture medium.

First, fluid dynamics within a Single-Flow MIVO^®^ device was investigated. The results show a capillary flow dynamic beneath the tissue cultured within inserts (Figure 4a); in particular, streamlines illustrating the flow field of the circulating medium in the basal chamber are associated with velocity values ranging from 0 to 1.5 cm/s, faithfully recapitulating capillary bloodstream when the imposed inlet flow rate is 2 mL/min. Subsequently, cisplatin mass transport analysis was carried out for 2D and 3D static and dynamic culture conditions (Figure 4b).

The maximal cisplatin percentages reached were lower in static conditions (64% and 95% for 3D and 2D, respectively) than in dynamic ones (100% for 3D and 2D). In static conditions, they were revealed to be higher in the 2D cell model than in the 3D sphere, whereas in dynamic conditions, no differences were noted. All curves present a constant increase in concentration until reaching a plateau value, which differs much more between the 3D static model than the 3D dynamic one. Moreover, the kinetics transport was very fast in all 2D scenarios and the 3D static one, whereas a slower rising over time developed in the 3D fluid-dynamic model.

#### 3.2.2. Cisplatin Toxo-Efficacy Evaluation in Static vs. Dynamic Conditions Models

**Figure 5:**
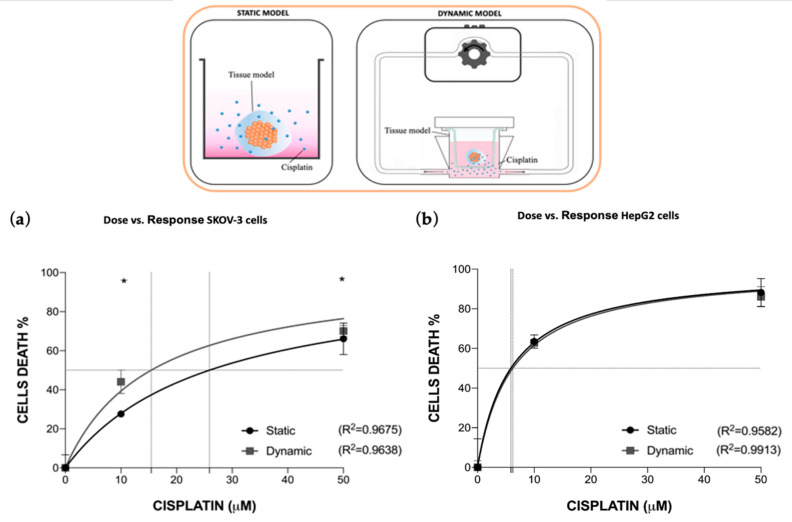
Dynamic culture condition impact on cisplatin pharmacodynamics in mono-culture models. Cisplatin dose-response of SKOV-3 (**a**) and HepG2 (**b**) cells under static or dynamic culture conditions. Values are reported as mean ± SD. The LD50 and EC50 parameters were calculated, with their R2. Statistical significance was set at * *p* < 0.05 (N = 3 biological replicates; *n* = 3 technical replicates).

The cytotoxicity of cisplatin was also investigated in the 2D liver cells model and 3D cancer cells model by introducing a physiological flow circulation, mimicking systemic drug administration. Data obtained by using the dynamic cell culture were compared with those obtained in static mono-culture conditions (Figure 5).

Interestingly, while in the 2D cellular model (i.e., liver) non-significant differences were noticed in LD50, cancer cells cultured within a 3D ECM displayed an effect of the drug that was significantly higher under flow, in line with the computational model. The EC50 parameter was revealed to be 25.9 μM and 15.4 μM for the static and dynamic culture conditions, respectively, meaning that fluid flow enhances drug diffusion through the 3D ECM-mimicking alginate hydrogel.

### 3.3. Multi-Organ-on-Chip Configuration Design and Development

**Figure 6:**
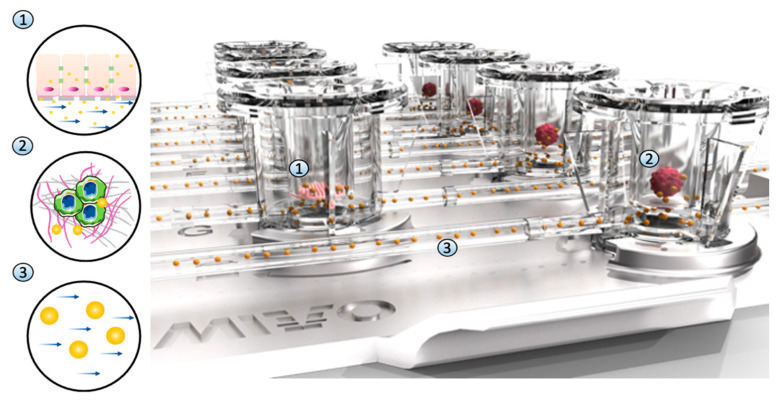
Dynamic multi-organ platform. A novel multi-organ-on-chip configuration was developed to co-culture HepG2 cells monolayers in one chamber (1) and ovarian cancer 3D hydrogel-based models in a second independent chamber (2) fluidically connected to the first one through an external fluidic circuit where cisplatin circulates (3), resembling the systemic drug administration.

A novel multi-organ OOC based on MIVO^®^ tissue chamber has been developed to allow physiological communications among different organs and simulate dynamic cisplatin systemic administration through an imposed capillary-like fluid flow (Figure 6). The OOC device was adopted since it allows both planar (2D) and 3D cell culture, being compliant with commercially available inserts. The luer-lock standard connectors of tubing guarantee a flexible and modular connection between different organs connected in series. The modular design of this OOC allows tissues to be previously prepared in static conditions and then moved into a dynamic cell culture. Moreover, the flexibility of this design and the adoption of fluidic standards enable a wide range of configurations tailored to the desired experimental setup: single vs. multi-organ configuration, the type and the number of cells, and shape and dimension of scaffolds where seeding cells, flow rate, and shear stresses are generated by the flow.

#### Cisplatin Toxo-Efficacy Evaluation in Single-Organ vs. Multi-Organ Conditions

The last experimental configuration achieved considered the possibility of simulating both cisplatin systemic administration through capillary blood flow conditioning and multi-organ culture by adopting the multi-compartmental setup of the MIVO^®^ organ-on-chip (Figure 6). Briefly, in one chamber the liver model was cultured onto a porous insert, while in a second chamber fluidically connected with the previous one, ovarian cancer tissue was 3D cultured. Data were compared with those obtained in co-culture static conditions.

**Figure 7:**
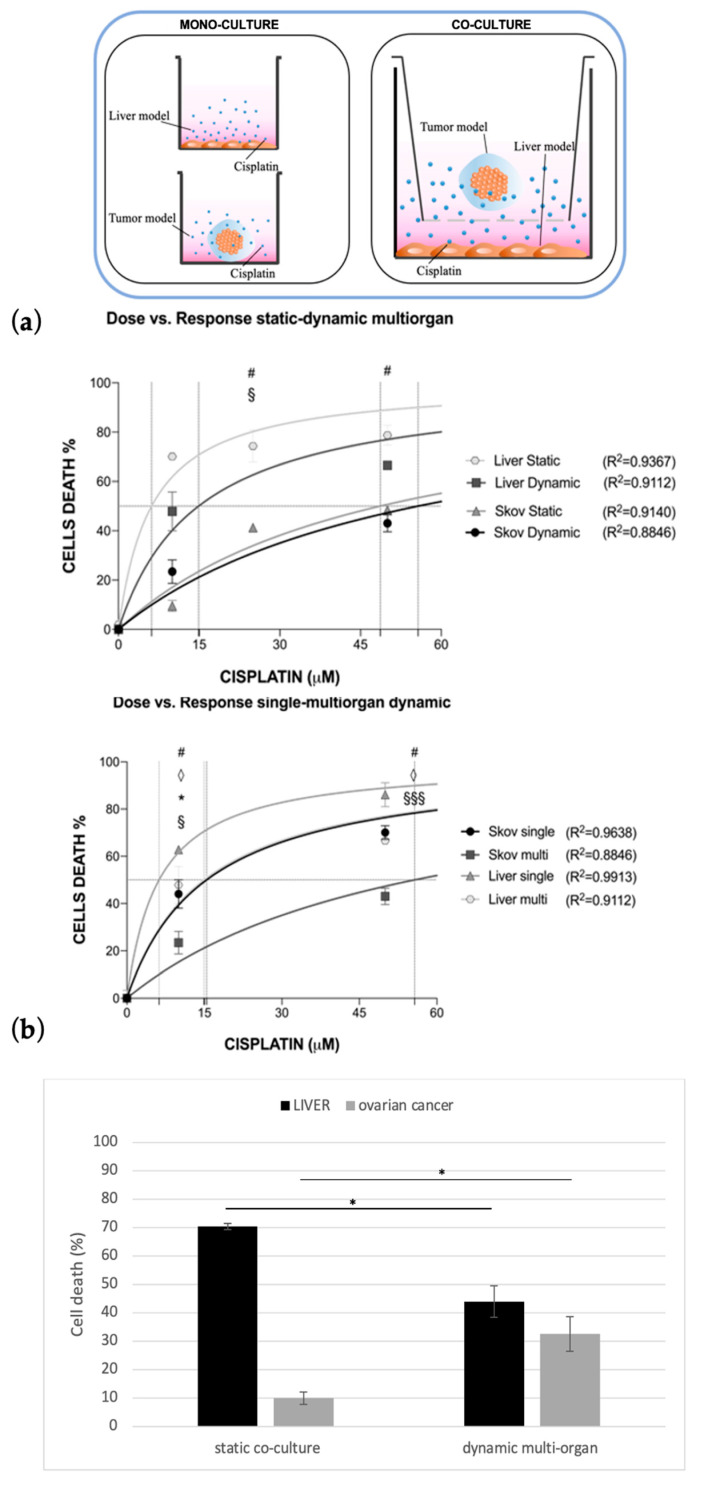
Cisplatin toxicity in single-organ vs. multi-organ conditions. Cisplatin dose-response of HepG2 and SKOV-3 cells when co-cultured in static vs. multi-organ dynamic conditions. Values are reported as mean ± SD. (**a**) The LD50 and EC50 parameter were calculated, with their R2; statistical significance was set at *, §, #, ◊ *p* < 0.05, §§§ *p* < 0.001. (**b**) The effect of cisplatin at plasma concentration was measured in both conditions for liver and ovarian cancer cells (N = 2 biological replicates; *n* = 3 technical replicates).

Cisplatin generated a significant dose-dependent inhibition in cell viability for both co-cultured cell lines, both in static and dynamic conditions (Figure 7a).

Interestingly, under plasmatic drug concentration (i.e., 10 μM), the hepatotoxicity was drastically reduced in dynamic multi-organ configuration, while the efficacy of the anticancer drug was shown to be significantly higher (Figure 7b).

## 4. Discussion

The development of new drugs is a complex and scientifically demanding task that is also highly time and cost-consuming. In addition, only a small number of the most promising new drug candidates successfully complete the clinical test phase and are released onto the market as approved drugs. To minimize this failure of potential candidates, the introduction of more predictive and significant test systems in the preclinical phase is becoming highly demanded. To this aim, several microfluidic OOC systems have been recently designed by introducing a fluid-dynamic environment to the cells in culture to recapitulate proper human cells behavior in vitro, such as in the human body, which is fundamental for a reliable prediction of pharmaco-kinetics and pharmaco-dynamics in subsequent clinical studies.

However, a great deal of work still has to be carried out to accelerate the adoption of these technological alternatives among pharma and CRO stakeholders. Indeed, OOCs are typically too difficult to be used than many other in vitro cell culture systems and their implementation needs highly specialized personnel, which can result in increasing experimental costs.

To avoid these issues, some of the authors have developed a highly flexible OOC device with the ability to grow established cellular models, both in monolayer and 3D matrixes, with different sizes and shapes. In particular, during its design, proper tuning of the chamber dimensions and culture media volume in circulation was achieved to guarantee the possibility of both culturing clinically relevant size tissues and sampling the sufficient amount of media volume for more robust and modular experimental readouts. Indeed, the use of miniaturized OOCs (i.e., few thousand of cells and a few microliters of media) does not allow tumors to be recreated to scale, and the microscale volume of these OOC amplify some adverse phenomena such as bubble formations and media evaporation, besides allowing the use of a small cellular range.

In previous work, authors reported a successful preclinical validation of an OOC-based drug efficacy assay in comparison with the xenograft model, which still represents the gold standard [17]. Here, a simultaneous toxo-efficacy assay has been achieved and successfully validated by co-culturing both liver and ovarian cancer tissue models in dynamic conditions, while cisplatin treatment was placed in circulation. Indeed, the unusual high modularity of this OOC makes both mono-culture and multi-organ configuration possible. The latter will allow multiple physiological niches to be built in separate chambers, where cells from different organs are cultivated under specific fluid flow-inducing shear stresses; during this multicompartmental cell culture condition, soluble molecule diffusion and cell–cell crosstalk can be successfully achieved through a serial fluidic connection among the chambers.

In particular, to promote a more in vivo-like environment for cancer cells, a 3D alginate hydrogel-based matrix with the proper stiffness and mechanical stability [51] was optimized in terms of cell viability, migration, and also molecule/drug diffusion. Although in this work, a human cell line was used to recapitulate the ovarian tumor model, this matrix supports the co-culture of multiple cells (e.g., immune cells and tumor cells) [20] and also the embedding of patient-derived cells spheroids (paper in preparation).

Moreover, a hepatoma cell line (HepG2) was used to mimic the liver tissue, since over the years it has represented one of the main standards in pharmacological and toxicological studies [52,53], retaining the different functionalities of the native organ [53], thus still being commonly adopted in innovative emerging pre-clinical platforms [52]. Nevertheless, the authors are aware that it lacks some features related to the metabolic activity of the human liver (e.g., low expression of cytochrome P450 enzyme) [54,55]. However, the use of HepG2 cells allowed us to perform a proper number of assays, which were fundamental to ensure the reproducibility of the experimental outcomes and to provide the proof of concept that the novel technological approach here described is a relevant pre-clinical tool. In fact, differently from primary hepatocytes, immortalized human hepatic cell lines are much more available and easier to manipulate and maintain in cell cultures for a prolonged time, besides being much cheaper without requiring any special operator skills [55]. Furthermore, although primary human hepatocytes more closely reproduce the phenotypic and functional aspects of the human tissue [56] as well as express many drug-metabolizing enzymes better reflecting the in vivo scenario [55], they are also characterized by high variability, due to their origin from various donors and the different isolation methods adopted among different groups, making the standardization of models highly challenging [55]; in addition, they retain the in vivo characteristics for short times when cultured in 2D traditional settings, often undergoing de-differentiating processes that could limit the reliability of the data [52,54,55,56].

Interestingly, while the importance of replacing 2D cell culture with 3D structures (with or without an extracellular component) to recapitulate the behavior of the cells closer to the in vivo scenario is widely accepted by scientists, the key role of dynamic flow conditions has still not been deciphered, especially when such 3D tissue models are adopted. Indeed, fluid flow resembles the proper feeding of tissue culture, which helps the tissue maintain a healthy state over time if compared to the static conditions [57], but, most importantly, it guarantees the proper diffusion of molecules (i.e., drugs) within the ECM as in vivo, thanks to our complex vasculature structure.

As also anticipated by the computational model, we have shown that only a residual part of the cisplatin is able to diffuse within the millimetric cancer tissue model cultured in vitro, and then to induce the expected cancer cells death. In fact, CFD showed that when a 2D model is adopted, the maximal drug concentration is rapidly reached with an over-estimated kinetic that does not recapitulate the in vivo scenario, where cells are not directly exposed to the drug treatment immediately after administration. Conversely, when a 3D static setting is adopted, the maximal percentage of cisplatin reaching cells is massively lower than in 2D models, since the ECM-like matrix limits the drug diffusion within the 3D structure. On the other hand, when the 3D tumor model is cultured in dynamic conditions, the slower and continuous rising of the diffusion up to 95% of the total amount better reproduces the drug delivery across the tissue, with a consequent increase in drug efficacy against the tumor as it happens in vivo.

Moreover, if the combinatory application of 3D tissue culture and fluid flow represents the necessary path to obtain more predictive preclinical outcomes, another revolutionary step to be taken into account is the simultaneous culture and drug treatment of multiple organs. From this perspective, we have adopted a multi-compartmentalized OOC to investigate and quantify the dose–response both in the on-target (e.g., cancer) and off-target (e.g., liver) organs, simultaneously. Compared to the static co-culture, the multi-organ OOC configuration allowed us to obtain a lower hepato-toxicity and higher efficacy of cisplatin against cancer cells by using a drug dose of 10 μM determined according to human plasmatic concentration.

Surprisingly, these results suggest that multi-organ and dynamic 3D culture are more predictive than the traditional static models, considering the wide use of this chemotherapeutic drug for treating women affected by ovarian cancer [58,59,60,61,62]. Moreover, it is worth discussing the experimental setting that we used in the dynamic multi-organ assay. In fact, liver cells were maintained as a monolayer instead of being cultured in a more complex and reliable model such as ovarian cancer cells. In this way, it was possible to highlight the role of any variable added step-by-step in these experiments (i.e., 3D culture, fluid motion, organ-organ connection). Furthermore, considering that hepatic cells cultured in 2D are more exposed to cisplatin than SKOV-3 cells, they should be basically more sensitive to the toxic effect of the drug; therefore, it is reasonable to conclude that by the decay of hepato-toxicity in such culture conditions, this experimental platform clearly demonstrates the importance of multi-organ connections in evaluating the systemic effects of chemical compounds.

## 5. Conclusions

In the present study, a multi-compartmental OOC platform was used to simulate systemic cisplatin administration, while 3D ovarian cancer tissues were fluidically connected to hepatic cellular models for studying therapeutic efficacy and hepato-toxic consequences in a physiological setting.

While the FDA is pushing the adoption of new alternative methods for regulatory use that can replace, reduce, and refine animal testing (3R) and improve the predictivity of non-clinical testing, this new in vitro validation of a toxo-efficacy assay represents another important brick towards the changes in 3R regulations. In this context, advancements in OOC technologies and their novel validations make it possible to test new therapies in a more predictive way and drastically reduce the use of animals in pre-clinical investigations. Similarly, the incorporation of 3D biology-inspired matrixes into a fluidic-based platform could result in the creation of next-generation tools for successful drug studies by evaluating new drug candidates and analyzing drug responses.

If the combination of 3D cancer models and fluidical stimuli resembling bloodstream circulation is necessary to develop a predictive pre-clinical platform that more closely reproduces what happens during therapy, a single-organ approach is not sufficient to determine the effective response to the drug treatment, both in terms of efficacy and toxicity. By using the MIVO^®^ fluidic device, it was possible to easily combine these three key aspects, firstly focusing on their single role, then studying what their response to the treatment when they are combined. Therefore, this system may represent a feasible alternative to the current pre-clinical settings that do not reflect the complexity of the human scenario, with the aim also to reduce animal testing according to the 3Rs principles.

Further studies on multi-organ clinically relevant-sized OOC might lead to a boost in the development of personalized therapies, in which toxicity and efficacy could be tested in vitro with highly robust, reproducible, and reliable technological approaches. Moreover, such a OOC-based experimental setup will also have a strong impact on basic research, improving the understanding of biological mechanisms behind systemic diseases.

## Data Availability

The data presented in this study are available on request from the corresponding author.

## Supplementary Materials

The following supporting information can be downloaded at: https://www.mdpi.com/article/10.3390/bioengineering10020270/s1, Figure S1: Cisplatin response in mono-culture vs. co-culture conditions (drug dose of 10 μM).
