# Supplementary material for: A Human Ovarian Tumor & Liver Organ-on-Chip for Simultaneous and More Predictive Toxo-Efficacy Assays
Source: Bioengineering (Basel). 2023 Feb 18;10(2):270. doi: 10.3390/bioengineering10020270 (PMC9952600; doi:10.3390/bioengineering10020270)
Supplement: Supplementary file 1 [file bioengineering-10-00270-s001.zip › bioengineering-2195291-supplementary.pdf]

Supplementary data

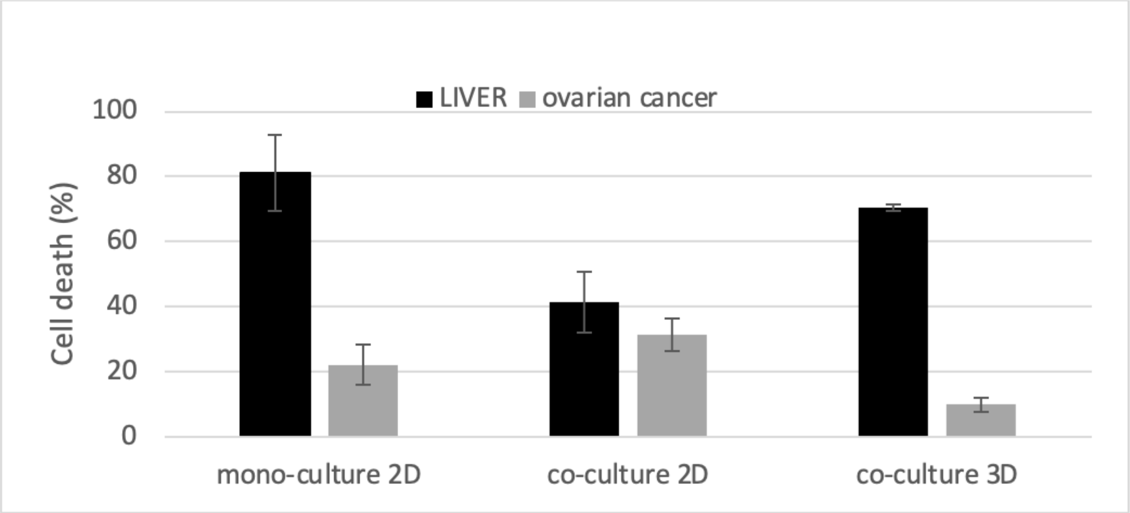

Figure S1: Cisplatin response in mono-culture vs co-culture conditions (drug dose of 10μM**).** The effect of cisplatin against liver and ovarian cells was measured considering a plasma concentration (i.e. 10μM). Values are reported as mean ± SD for each condition.
